# Microglial Corpse Clearance: Lessons From Macrophages

**DOI:** 10.3389/fimmu.2020.00506

**Published:** 2020-03-27

**Authors:** Mar Márquez-Ropero, Eva Benito, Ainhoa Plaza-Zabala, Amanda Sierra

**Affiliations:** ^1^Achucarro Basque Center for Neuroscience, Parque Científico, University of the Basque Country (UPV/EHU), Leioa, Spain; ^2^Department of Neuroscience, University of the Basque Country (UPV/EHU), Leioa, Spain; ^3^Ikerbasque Foundation, Bilbao, Spain

**Keywords:** microglia, macrophages, phagocytosis, apoptosis, efferocytosis, epigenetic, metabolism, trained immunity

## Abstract

From development to aging and disease, the brain parenchyma is under the constant threat of debris accumulation, in the form of dead cells and protein aggregates. To prevent garbage buildup, the brain is equipped with efficient phagocytes: the microglia. Microglia are similar, but not identical to other tissue macrophages, and in this review, we will first summarize the differences in the origin, lineage and population maintenance of microglia and macrophages. Then, we will discuss several principles that govern macrophage phagocytosis of apoptotic cells (efferocytosis), including the existence of redundant recognition mechanisms (“find-me” and “eat-me”) that lead to a tight coupling between apoptosis and phagocytosis. We will then describe that resulting from engulfment and degradation of apoptotic cargo, phagocytes undergo an epigenetic, transcriptional and metabolic rewiring that leads to trained immunity, and discuss its relevance for microglia and brain function. In summary, we will show that neuroimmunologists can learn many lessons from the well-developed field of macrophage phagocytosis biology.

## Introduction

Microglial phagocytosis of apoptotic cells (efferocytosis) is at the core of the brain regenerative response. Its relevance in maintaining brain tissue homeostasis from development to aging and neurodegenerative diseases is undisputed but, nonetheless, many fundamental questions about the biology of the process remain open: How is phagocytosis efficiency regulated at the cellular and molecular levels? How can it be manipulated? Is it a dead-end road or does it trigger changes in the phagocyte? Is phagocytosis simply a process of garbage removal or does it actively participate in the well-being of the surrounding tissue? We here try to address these questions by collecting answers from microglia's cousins, the macrophages that reside in other tissues. In the first part of this review, we will discuss similarities and differences in the identity of microglia and other macrophages, taking into account their developmental origin and the maintenance of the adult populations. In the second part, we will compare the mechanisms that mediate recognition and engulfment and their epigenetic, transcriptional, metabolic, and immunological consequences. We will conclude that phagocytosis has a tremendous potential to impact on brain physiology and pathology.

## Lineage and Origins of Microglia and Other Macrophages

Microglia are brain-resident macrophages. They interact with the brain parenchyma and carry out essential maintenance functions. They are often studied independently from other tissue-resident macrophages, probably because they are unique in some aspects, most notably in their isolation from the rest of the body through the blood brain barrier (BBB). But how different are microglia really from other tissue resident macrophages in terms of origin, lineage, and identity? A close review of the literature shows that microglia are not as coarsely distinct to other macrophages as one may think, yet there are some fine differences in how they behave in their local environment. In the next sections, we will review evidence about the origin, lineage, identity, and population dynamics of microglia compared to other tissue-resident macrophages and highlight commonalities and differences.

### Lesson 1. The Monocytic Origin of Macrophages Is More the Exception Than the Rule

Macrophages are widespread and reside in many different organs, where they fulfill different functions. Because it is such a diverse population of cells, a fundamental question is whether they have a common precursor or whether each macrophage population develops from a different precursor. Based on the observation that some macrophages are short-lived and that they are often renewed by circulating monocytes, already in 1972 van Furth et al. proposed the “mononuclear phagocyte system” theory, by which tissue-resident macrophages were assumed to derive from blood-circulating monocytes and to differentiate within the host tissue (1). However, at that time there was evidence that some macrophage populations can proliferate locally and self-renew (2, 3), which clashed with the idea that macrophages are of monocytic origin. Why would they need to divide and self-renew locally if the main source is in fact a pool of circulating monocytes? Over the last decade, tracing and parabiosis experiments, where the origin and location of specific macrophage populations can be followed over time, have demonstrated that in fact most macrophage populations originate in the embryo prenatally. And it is only some macrophage populations, such as gut (4), dermis (5), pancreas (6), and heart (7) macrophages that are replaced from circulating monocytes. In contrast, other macrophage populations, most notably microglia and skin macrophages (Langerhans cells), but also others, originate early during development, are long-lived and capable of local self-renewal with no significant involvement of circulating monocytes (8–12).

**Figure 1:**
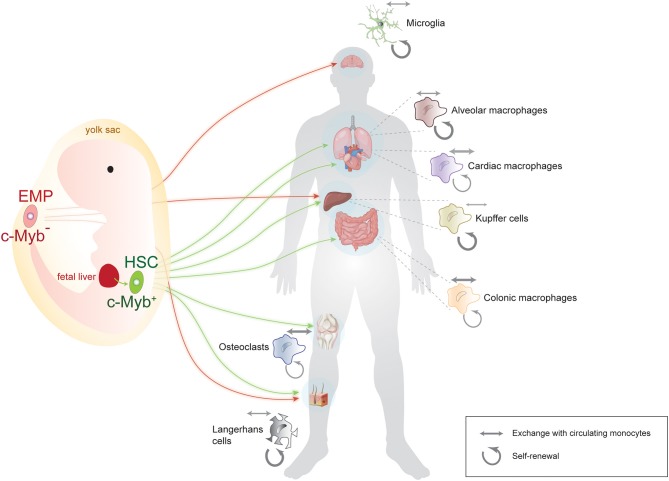
Summary of the origin, lineage, and population dynamics of main macrophage populations. Some macrophages, such as microglia, originate exclusively from an early, c-Myb-negative erythromyeloid precursor (EMP) in the yolk sac, while others, such as Langerhans and Kupffer cells, have a mixed yolk sac-fetal liver origin. Many macrophage populations originate from c-Myb-positive hematopoietic stem cells (HSC) after traveling through the main prenatal hematopoietic location, the fetal liver. Note that for the sake of simplicity, arrows indicate the main site of origin of macrophages, although for some HSC-derived macrophage populations, some contribution from the yolk sac has also been reported. Weighted arrows next to each tissue-specific macrophage population indicate the relative contribution of self-renewal (circular arrows) vs. exchange with circulating monocytes (double-sided arrows) to that particular population. Microglia and Langerhans cells for instance are mainly self-renewing, whereas cardiac and colonic macrophages do exchange significantly with circulating monocytes. Some vector graphics were obtained from Vecteezy.com with permission.

The notion that macrophages have a prenatal and not a monocytic origin caused a paradigm shift in the field and prompted questions about their precise site of origin and about the routes by which they reach target organs. The precise spatial origin of each macrophage pool is still under debate, but there are essentially two accepted sources: the yolk sac and the aorta-gonad mesonephros. From there, macrophages take one of two routes: they migrate directly to their target organ or they go through fetal liver and then on to their target tissue (Figure 1). What percentage of the mature tissue-resident macrophages in fact originate via each of these routes is still being investigated for most macrophage populations. In this respect, the origin of microglia is probably the most undisputable: they originate in the yolk sac and migrate directly to the brain as early as embryonic day E10.5 in the mouse. Other macrophages, such as Langerhans cells seem to have a mixed origin: some derive directly from the yolk sac, whereas others travel through the fetal liver before they reach their destination (9). The latter route through fetal liver seems to be the standard and more prevalent for most other macrophage populations (13, 14).

In summary, the original theory that tissue-resident macrophages universally derive from circulating monocytes has now been replaced with a more refined picture, where macrophages in fact originate from an early embryonic precursor, seed their target tissue, and self-renew locally (12). In this new picture, macrophages that derive from circulating monocytes are more the exception than the rule and microglia probably constitute the paradigmatic example of macrophages of prenatal origin with self-renewal capacity and no exchange with circulating monocytes under physiological conditions.

But if monocytes are not the precursor cells for most macrophages, what is in fact the molecular identity of their precursors and how different is it for different macrophage populations? In the next section, we will summarize what is known about the identity of the embryonic precursors that give rise to different tissue-resident macrophages.

### Lesson 2. Microglia Do Not Require the Transcription Factor c-Myb to Develop, but Other Macrophages Do

After it was demonstrated that most macrophages originate prenatally either from the yolk sac or from the aorta-gonad mesonephros, investigators turned their attention to the precise molecular identity of the precursors. The goal was to identify markers and transcription factors that would allow us to identify and classify them.

There is a certain correlation between the physical and temporal origin of macrophages and the markers they express. The most polarizing marker, the one that probably allows the most straightforward classification, is the transcription factor c-Myb. Early (E7.5-8.5 in mice) progenitors from the yolk sac (erythromyeloid precursors or EMPs) do not express or require the transcription factor c-Myb for development and maturation. Accordingly, macrophages that stem from EMPs, such as microglia, Langerhans cells and Kupffer cells (liver macrophages), develop normally in c-Myb^−/−^ animals (10). In contrast, late (E10.5 in mice) progenitors that originate in the fetal liver (hematopoietic stem cells, HSCs) do express and require c-Myb for proper development (15, 16). This is the case for most tissue-resident macrophages, which fail to develop in the absence of c-Myb (10). c-Myb dependence is therefore a classifying criterion for macrophage lineage, but it is not the only transcription factor involved in maturation. The overall topic of macrophage lineage markers is beyond the scope of this review and has been covered extensively before (13). For microglia in particular, key players in their development include the growth factor receptor CSF1R (Colony Stimulating Factor 1 Receptor), the chemokine receptor CX_3_CR1 (CX3C chemokine receptor 1), the calcium binding protein Iba-1 (ionized calcium binding adaptor molecule 1), the G-protein-coupled receptor F4/80 (also known as EMR_1_ - EGF-like module-containing mucin-like hormone receptor-like 1) and the integrin CD11b (cluster of differentiation molecule 11B, also known as Integrin Alpha M - ITGAM) (17, 18).

The lesson that derives from these studies is that ontogeny and molecular identity of different macrophage populations go hand in hand. There is an additional component, longevity, which also shows some level of correlation with macrophage ontogeny. Early, c-Myb-negative progenitors in the yolk sac give rise to populations that tend to be long-lasting (microglia, Langerhans and Kupffer cells), and later, c-Myb-positive cells in the mesonephros and fetal liver give rise to a mix of long- and shorter-lived populations. Microglia are particularly long-lived and have the ability to self-renew several times over a lifetime (19). A question that derives from this notion is how the population behaves with respect to individual cells. What is the natural “life cycle” of a single microglia and how does it contribute to the overall stability of the population? The next section will examine evidence of how microglia are replenished under homeostatic conditions.

### Lesson 3. As Long as the Blood-Brain Barrier Is Intact, Microglia Can Self-Renew Throughout a Lifetime Without a Significant Monocyte Contribution

As reviewed in the previous section, under physiological conditions, microglia have a prenatal yolk sac origin. The BBB protects and isolates the brain from exchange with blood circulation, effectively turning it into a very isolated microenvironment. Here, microglia stay stable and self-renew with virtually no contribution from circulating monocytes in physiological conditions (8). But what is the exact population dynamics of microglia and how do they behave individually? And what happens if microglia are depleted in a healthy brain, how do they replenish under these conditions?

One critical factor in microglial development and survival is CSFR1. Mice devoid of this receptor do not have microglia (8, 20), and mutations that affect the function of the kinase domain of the receptor cause severe neurological syndromes in mice and humans (21–24). In line with this, in 2014, Elmore et al. showed that a chemical inhibitor of CSF1R virtually ablated the microglial population in the healthy brain. Strikingly, they also showed that microglia could be repopulated upon withdrawal of the inhibitor (25). It was then critical to identify which cells microglia repopulated from. The original report suggested the existence of a “hidden” microglial progenitor expressing the intermediate filament nestin (25). However, more detailed studies have in fact demonstrated that the most likely scenario is one where microglia replenish from the few remaining cells that are not removed during CSF1R inhibition (19, 26). A few questions are left unanswered regarding how this repopulation happens, including why some cells remain resistant to CSF1R inhibition and what signaling mechanisms tell microglia that they need to stop proliferating once the repopulation is completed. In this regard, it is interesting to note that microglia seem to have a very active population dynamics at steady-state, with a balanced ratio of proliferation and apoptosis and several self-renewal cycles during a lifetime (19).

A similar scenario is observed in Langerhans cells, which also have a predominant yolk sac origin, are long-lived (9), renew during a lifetime (27) and most likely proliferate from fully differentiated cells (28). Indeed, proliferating, self-renewing macrophages can be found in almost all tissues under physiological conditions (11, 29), and often even more so under pathological conditions, although in the latter case the contribution from circulating monocytes is most likely instrumental (30, 31). Only when the BBB integrity is compromised, most commonly after head irradiation, circulating monocytes can give rise to functional microglia [for a review see (31)]. Nonetheless, the fact that a population of cells exists that has the potential to differentiate into microglia under certain pathological conditions is highly relevant for therapeutic intervention and merits further research.

Overall, macrophages seem to be in charge of their own local population dynamics and reach a steady state of functional self-renewal with balanced division/death rates that match the functions they carry out in their host tissues.

### Lesson 4. Microglia Have Specialized in Homeostatic and Stimulus-Triggered Remodeling of Brain Circuits and Structure

The original notion that macrophages are a uniform pool of phagocytes that reside in all organs and perform immune functions has long been discarded. Instead, the current view is that macrophages are essentially as dissimilar as their target tissues and have specialized in performing tissue homeostasis functions that make sense in their local microenvironment. For instance, whereas lung macrophages are specialized in surfactant clearance (32) and adipose macrophages participate in lipid and insulin metabolism (33), spleen macrophages are critical for iron homeostasis (34, 35). In the particular case of microglia, their specialized functions include interactions with neurons, potentially participating in synaptic remodeling (36), modulating experience-triggered events, such as neurogenesis (37), and participating in memory acquisition (38, 39). Microglia are also very active in surveilling the brain parenchyma with their highly motile processes (40, 41) and this is believed to be essential for brain maintenance and protection and to be in close connection with neuronal activity (42, 43). However, microglial motility may not be so unique, as late evidence suggests that other macrophages might also perform this type of surveillance in the intersticial space (44).

It is not surprising that macrophages have specialized roles within their host tissues to aid their homeostasis and maintenance. In this context, microglia need to be in close contact with neurons and be highly sensitive to changes in the microenvironment. In the next section, we will focus on this sensitivity to environmental factors and how that may impact the epigenetic and transcriptional identity of microglia.

### Lesson 5. Microglia Have Developed a Unique Transcriptional and Epigenetic Landscape

Given the wide diversity of macrophage tissue-specific phenotypes, a very relevant question to ask is how different macrophages are at the molecular level. How different are their transcription and epigenetic profiles under physiological conditions and how do they respond to changes in their environment? With the advent of “omics” methods and, in particular, with the application of single-cell studies the field has experimented a boom. It is now clear that macrophages are as different at the transcription and epigenetic level as they are at the phenotypic level. Macrophages share a common core transcriptional identity, but each tissue-specific population expresses a unique set of transcripts that is organ-specific (45, 46). In line with this, microglia are transcriptionally distinct to other macrophage populations but, in addition, single-cell studies have shown that there are several subpopulations of microglia that could perform functionally different tasks under basal and inflammatory conditions (47, 48). Similar observations are being made in other macrophage populations, with single-cell omics techniques revealing their underlying richness and diversity (49, 50).

In the particular case of microglia, these studies have revealed that microglia are transcriptionally homogeneous across brain regions but diverge in their transcriptional profiles across their cell division state (48). Another distinct microglia subpopulation that has earned a fair bit of attention is the so-called Disease-Associated Microglia (DAM), a subgroup of microglia revealed by single-cell sequencing specifically in the brain of an Alzheimer's disease mouse model (47). However, despite the power of these approaches in revealing the heterogeneity of distinct microglia subpopulations, there is debate as to the relevance of these populations in human brain, which seem to have subpopulations of their own (see comment in https://www.alzforum.org/news/research-news/when-it-comes-alzheimers-disease-do-human-microglia-even-give-dam). Ultimately, and under the premise that to study human disease one should focus on human microglia as much as possible, these studies reveal that microglia are in fact more diverse than anticipated and encourage further investigation aiming at dissecting their identity.

An ever-expanding source of cell diversity lies in the epigenetic landscape, which is most commonly read in the form of histone modifications (acetylations, methylations), DNA modifications (DNA methylation) and small non-coding RNAs (miRNAs, lncRNAs), and which has also been probed in different macrophage populations. The combinatorial patterns of histone 3 acetylation (at lysine 27) and methylation (at lysine 4) have been profiled in detail, revealing a core epigenetic macrophage signature, as well as a set of specific enhancer marks that are unique to each organ (46). This study also served to validate the idea that local macrophage identity is shaped by the local microenvironment. Previous experiments had shown that some macrophages populations are highly plastic and can adopt a tissue-specific identity even after ex vivo culture (51, 52). Recent evidence suggests that this phenotypic identity reshaping is accompanied by rewiring of the epigenetic landscape (46). In this context, microglia proved to be transcriptionally and epigenetically distinct to all other tissue-resident macrophages and clustered systematically separated from other populations for different epigenetic marks (46).

A follow-up question that emerges from these observations is to which extent epigenetic reprogramming is relevant for macrophage biology, and how it may affect microglia in particular. Microglia depletion studies in diseased brains have shown that after replenishment with “fresh” microglia, animals have improved cognitive scores (53–55). These studies have played with the idea that microglia in a diseased brain may have an altered epigenetic signature that is erased at the time of depletion and that the replenishing microglia are reprogrammed and therefore functionally fitter. How much of the original epigenetic identity is retained by the few remaining microglia and what role exactly cellular reprogramming plays in microglia phenotype is still unclear. Nonetheless, microglia and other tissue-resident macrophages appear to have a unique transcriptional and epigenetic signature that is highly plastic and may play a fundamental role in how they interact with their environment.

Since the molecular and phenotypic identities of any cell are in constant interplay, it is expected that different macrophages with different tissue-specific functions and phenotypes would have different transcriptional and epigenetic signatures. A more thorough characterization of the effect of environmental factors on cellular reprogramming and function will shed light on how dynamic these changes are and, most importantly, how relevant under physiological and disease situations. However, in spite of their phenotypic diversity, macrophages do share some core functions, such as phagocytic clearance of dead cells and immune signaling. Yet even those have tissue-specific nuances. The next section will address the similarities and disparities in mechanisms and dynamics of phagocytosis in microglia vs. other macrophages.

## Phagocytosis by Macrophages and Microglia

The efficient phagocytic removal of apoptotic debris (efferocytosis), mostly carried out by resident macrophages, vastly influences our daily physiology. It is estimated that billions of cells are removed everyday (56): aged erythrocytes and neutrophils in the spleen, liver and bone marrow (57, 58), epithelial cells in the mammary gland after the lactation period (59), spermatogenic cells in the testis (60), and the outer segment of light-exposed photoreceptors in the retina (61), among others. In the brain, microglia remove the excess newborn cells produced during embryonic and postnatal development in the cortex, cerebellum and amygdala (62–64) and in adult neurogenic niches in the hippocampus and subventricular zone (SVZ) (37, 65), as well as deceased cells during aging and neurodegenerative diseases (66). Other cell types, such as astrocytes, neuroblasts or cells of the neural crest may also act as phagocytes but generally with less efficiency and thus microglia are considered the brain “professional” phagocytes (67).

In this section, we will compare available data on the process of phagocytosis by macrophages and microglia. We will then describe that phagocytes express overlapping recognition mechanisms of apoptotic cells, and that this redundancy ensures that phagocytosis is fast and efficient. We will then describe the functional consequences of ingestion in the phagocyte, focusing on inflammatory responses, and metabolic adaptations. Ultimately, we will conclude that phagocytosis is a powerful mechanism that needs to be firmly controlled to ensure the efficient removal of target cells while preventing the demise of live cells.

### Lesson 6. Phagocytosis Is Tightly Coupled to Apoptosis Due to Redundant Recognition Mechanisms

Phagocytosis and apoptosis are evolutionarily linked together as a mechanism that allows the elimination of excess, dysfunctional, or aged cells without imposing alterations or damage in the surrounding cells (67). Homeostatic phagocytosis is ensured by the immediate recognition of the apoptotic cell by phagocytes through a redundant plethora of released “find-me” and membrane-bound “eat-me” signals that are recognized by the phagocytes (Table 1). Herein we summarize some of the most relevant signals and receptors involved in apoptotic cell recognition, and we prompt the reader to comprehensive reviews on the topic for more details (67, 72).

**Table 1:** Expression of phagocytosis-related receptors by microglia.

**Phagocyte receptor**	**Gene**	**Function**	**Brain database (68)**	**Immunological database (skyline)**	**Human database (69)**	**DAM microglia (47)**	**Aging human signature (70)**	**Aging human database (70) vs. (68)**	**Cerebellum vs. Striatum (71)**	**PAM microglia (48)**
G2A	GPR132	G protein-coupled receptor (GPCR), recognizes lysophosphatidylcholine	Microglia	T lymphocytes, DCs. Neurotrophils	Mo > Mi =Ma	-	HuMi_Aged	Up	-	-
CX3CR1	CX3CR1	Fractalkine receptor	Microglia	Microglia (Monocytes low)	Mi >> Mo >> Ma	Down	HuMi_Aged	Down	Striatal	Down
P2Y6	P2RY6	Purinergic receptor type Y6	Microglia	Microglia (Macrophages low)	Mi = Ma > Mo	Down	HuMi_Aged	Up	Striatal=Cerebellar	=
P2Y12	P2RY12	Purinergic receptor type Y12	Microglia (OPCs low)	Microglia	Mi >> Mo >> Ma	Down	HuMi_Aged	Down	Striatal	Down
BAI1	BAI1 (ADGRB)	adhesion G protein-coupled receptor B1	Astrocytes, neurons, OPCs, oligodendrocytes	Epithelial cells, neutrophils, NKs	Mi = Ma = Mo	-	-	-	-	-
TIM4	TIMD4	T cell immunoglobulin and mucin domain containing 4	Microglia	Macrophages	-	-	-	-	Cerebellar	-
Stabilin 1	STAB1	-	Microglia (endothelium low)	Microglia, endothelium	Mi = Ma > Mo	Down	HuMi_Aged	Up	-	Up
Stabilin 2	STAB2	-	Endothelium	Endothelium, macrophages	-	-	-	-	-	-
SIRPa	SIRPA	Signal regulatory protein alpha	Microglia	Microglia, macrophages, neutrophils	Mi = Ma = Mo	Down	-	Down	Striatal	-
CD300b	CD300LB	CD300 molecule like family member B	Microglia	Neurotrophils, macrophages	Mo = Ma >> Mi	-	-	-	-	-
TREM2	TREM2	Triggering receptor expressed on myeloid cells 2	Microglia	Microglia	Mi > Ma > Mo	Up	HuMi_Aged	Up	-	Up
MerTK	MERTK	MER proto-oncogene, tyrosine kinase	Astrocytes, microglia	Microglia, macrophages	Mi >Ma > Mo	Down	-	Down	Striatal	-
Axl	AXL	AXL receptor tyrosine kinase	Astrocytes, OPCs	Macrophages	Mi > Mo > Ma	Up	HuMi_Aged	No change	Cerebellar	-
Tyro3	TYRO3	TYRO3 protein tyrosine kinase	Oligodendrocytes	NKs, T lymphocytes	Mi = Ma = Mo	-	-	-	-	-
CD36	CD36	Fatty acid translocase	Astrocytes, OPCs, microglia	macrophages, endothelium	Ma = Mo >> Mi	-	-	-	-	-
CD11b	ITGAM	Integrin subunit alpha M	Microglia	Neurotrophils, macrophages, microglia	Mi = Ma = Mo	Down	HuMi_Aged	No change	Striatal	-
CD206	MRC1	Mannose receptor C-Type 1	Microglia	Macrophages	Ma > Mi > Mo	-	HuMi_Aged	Down	Cerebellar	-
Clec7a	CLEC7A	C-type lectin domain containing 7A	Microglia	Macrophages, neutrophils, monocytes	Mo > Ma > Mi	Up	HuMi_Aged	Down	-	Up
CD22	CD22	Sialic acid-binding Ig-like lectin 2	Microglia	Macrophages	Ma > Mo > Mi	Up	-	No change	Striatal	-

*The table indicates the name of the receptor, the gene ID, its function and the expression in different cell types found in RNAseq public databases: a brain-specific database of microglia compared to other brain cells (68); an immunological database of microglia and other immune cells (http://rstats.immgen.org/Skyline/skyline.html); a human database of microglia (Mi), macrophages (Ma), and monocytes (Mo) (69); a database of genes upregulated (Up) and downregulated (Down) in disease-associated microglia (DAM) (47); a database of genes specific of a human aging signature (HuMi-aged) (70); a database of genes enriched in cerebellar (non-phagocytic) and striatal (phagocytic) microglia (71); and a database of genes upregulated (Up) or downregulated (Down) proliferative-region associated microglia (PAM)(48)*.

Among “find-me” signals stand out purines (ATP, UTP, and their dephosphorylated derivatives) and the chemokine fractalkine, and the corresponding metabotropic purinergic P2Y receptors and CX3CR1 expressed in phagocytes. Another important “find-me” signal is the lipid lysophosphatidylcholine (LPC), which binds to GPR132 (G2A) receptors in phagocytes. The best known “eat-me” signal is the lipid phosphatidylserine (PS), with its many phagocyte receptors, such as BAI1, TIM4 and stabilins. PS is also indirectly recognized by several bridge-molecules, including MFG-E8 (Milk-fat globule E8), as well as Protein S and Gas6, which bind to members of the TAM (Tyro3, Axl, Mer) family in phagocytes. In addition, other well-known receptors classically involved in immune responses are the inflammation triggering receptors expressed on myeloid cells TREM2 and CD300B; and integrins and complement receptors such as CD11b, which recognize opsonized apoptotic cells both directly and some of them indirectly via MFG-E8. More recently, three sugar-related receptors have been involved in phagocytosis: the mannose receptor CD206 (MRC1), which is upregulated in bone marrow and intestinal phagocytes compared to non-phagocytic macrophages and predicts their phagocytic performance (73). The sialic acid binding protein CD22, a negative regulator of phagocytosis identified in an *in vitro* screen that is upregulated in microglia in the aging brain (74). And the beta glucan receptor Clec7a, associated to the phagocytosis of oligodendrocytes by microglia during early postnatal development (48). Each type of phagocyte expresses their own set of receptors (67) and microglia is no exception.

The most conspicuous phagocytosis receptors expressed in both mouse and human microglia are CX3CR1, P2Y6, P2Y12, stabilin 1, SIRPα, TREM2, MerTK, and CD11b, based on brain RNASeq databases (68, 69) and the immunological RNASeq database Skyline (http://rstats.immgen.org/Skyline/skyline.html) (Table 1). However, it is important to note that each of these receptors is dynamically expressed during aging, disease and in different brain areas (47, 48, 70, 71) (Table 1), and each may serve different functions. For instance, in macrophages, MerTK and Axl show opposite regulation of their expression during inflammatory conditions, and specialize in different types of phagocytosis: MerTK in homeostasis, Axl during inflammation (75). Similarly, CD206, MerTK, and TIM4 (but not other receptors) are upregulated in peripheral macrophages early upon phagocytosis (73). In microglia, the expression of these receptors is not a proxy of their phagocytic performance either, as some including CX3CR1, P2Y12, and MerTK, have higher expression in striatal than in cerebellar microglia, whereas phagocytosis seems more prominent in the cerebellum than in the striatum (71). Similarly, their expression is also not correlated with PAM (proliferative-region- associated microglia), which has been involved in phagocytosis of developing oligodendrocytes during early postnatal development (48). Moreover, the efficiency of phagocytosis does not rely on their expression alone, but also on the motility of the microglial processes, the relative distribution of microglia compared to apoptotic cells, or the lysosomal efficiency, to name a few other factors. Their expression, however, has been linked to functional changes in microglia. For instance, some of these receptors, such as CX3CR1 and P2Y12, are part of the so-called “homeostatic microglia signature,” and are downregulated in DAM (47) but upregulated in aging microglia (70). Therefore, the expression of phagocytosis receptors is not invariably linked to microglial phagocytosis efficiency. A careful analysis of the subsets of receptors expressed by microglia in different regions across the lifespan and during specific disease will shed light into their functional implication in microglial phagocytosis.

Finally, as microglia is specialized in surveilling the brain parenchyma, several receptor systems involved in the recognition of apoptotic cells have also been “hitchhiked” by other brain-specific cargos. For instance, TREM2 has been deeply involved in the recognition of beta amyloid extracellular deposits in the context of Alzheimer's disease (AD) (76), and is one of the main genetic risk factors for its development (77). The complement receptor CD11b (78, 79) and the fractalkine receptor CX3CR1 (36) are used to recognize dendritic spines and are related to synaptic prunning. SIRPα recognizes the “don't-eat-me” signal CD47 on myelin debris and on active synapses, inhibiting their phagocytosis (80, 81). CD22 binding to sialic acid inhibits the phagocytosis of extracellular deposits of beta amyloid, myelin and α-synuclein during aging (74). However, to which extent the phagocytosis of beta amyloid, α-synuclein, spines, and synapses, or myelin debris phenocopies the complete process of efferocytosis, including attraction, engulfment, and degradation, remains to be determined.

### Lesson 7. Phagocytosis Is Fast and Apoptosis Is Silent

A consequence of the tight coupling between apoptosis and phagocytosis is that phagocytes are not easily caught red-handed and, consequently, apoptosis is largely underestimated. Original experiments in the thymus, where T lymphocytes undergo chromosomic rearrangement in their antigen receptor genes, suggested that the negatively selected cells were removed but few apoptotic cells were found. It was only when phagocytosis was analyzed that it became evident that apoptotic T lymphocytes were quickly removed by resident macrophages (82), leading to the suggestion that phagocytosis lasts minutes (72). Similarly, taking into account the number of erythrocytes removed daily in the spleen and liver, and the number of resident macrophages in these tissues (pulp macrophages and Kupffer cells, respectively), phagocytosis has been estimated to last under 30 min (67). Microglia are comparably fast. Using as a model the adult neurogenic cascade of the hippocampus, where newborn cells naturally undergo apoptosis and are phagocytosed by microglia, the estimated average clearance time of an apoptotic cell is 90 min (37).

**Figure 2:**
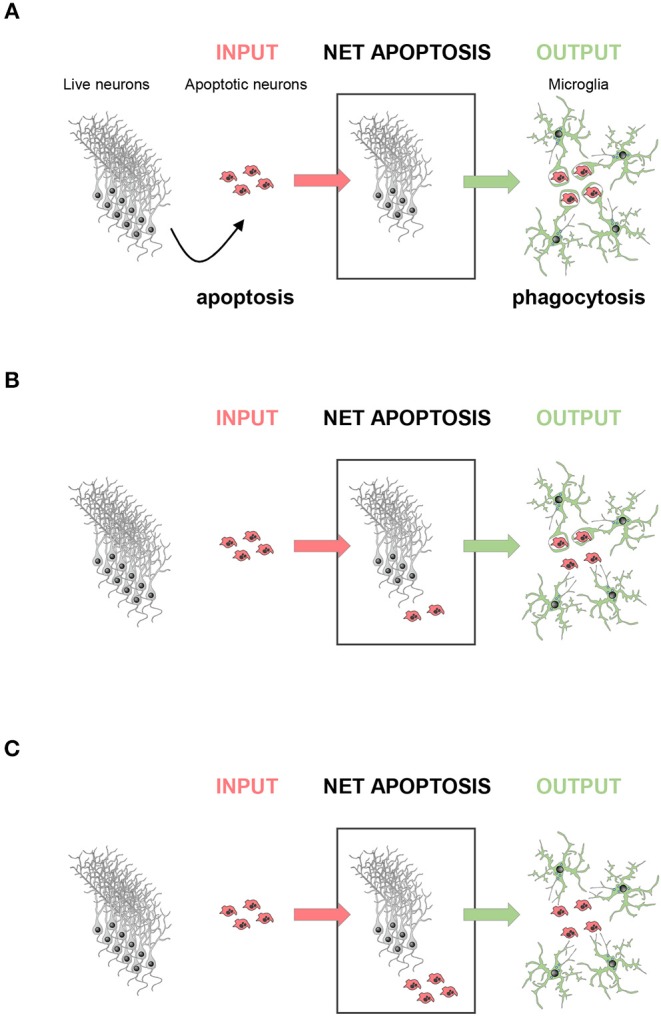
The amount of apoptosis visualized depends on phagocytosis efficiency. The net apoptotic cell number observed (net apoptosis) at a given time point depends on the rate of live neurons undergoing apoptosis (input) and the rate of clearance of the apoptotic cells by microglia (output). **(A)** In a perfect scenario, the rate of phagocytosis would be comparable to the rate of apoptosis induction, and no apoptotic cells should be observed. **(B)** In most cases, apoptosis induction is faster than engulfment and degradation, and only some apoptotic cells would be observed, whereas others are already cleared by microglia. **(C)** In a dysfunctional scenario with deficient phagocytosis, all cells undergoing apoptosis would be visible.

A corollary of this data is that phagocytosis efficiency determines the amount of apoptosis visualized. At a given time, the number of apoptotic cells found can be conceived as a black box with doors on each side: an incoming door that represents the cells that enter apoptosis *de novo*; and an outgoing door that represents the cells that are removed via phagocytosis. Using this analogy is easy to understand that the size of the pool of apoptotic cells depends on the relative velocities of the two processes, apoptosis and phagocytosis (Figure 2). Therefore, in physiological conditions apoptotic cells are difficult to observe because microglial phagocytosis is very efficient (37). In contrast, in pathologies like epilepsy, the increased number of apoptotic cells in early stages is not due to apoptosis induction, but to phagocytosis impairment and accumulation of non-removed apoptotic cells (42). In conclusion, phagocytosis efficiency determines the dynamics of apoptosis during development and disease.

### Lesson 8. Phagocytosis Is Immunomodulatory at the Epigenetic, Transcriptional and Posttranslational Levels

The tight coupling between apoptosis and phagocytosis has functional relevance, as it prevents the release of intracellular contents in physiological conditions. In contrast, during trauma, uncontrolled and unexpected cell death is usually followed by infection by microorganisms, and the dead cells release damage-associated molecular patterns (DAMPs), such as DNA, RNA, nucleotides, or chromatin proteins such as HMGB1 (high mobility group box 1 protein). These signals are initially recognized by the fluidic branch of the innate immune response (i.e., the complement and the coagulation systems), followed by recognition of specific pattern recognition receptors (PRRs) in several types of leukocytes. This cascade of events initiates a complex immune response that includes chemokine and cytokine release, release of reactive oxygen species and other responses to heal the damaged tissue and kill invading microorganisms (83). Unlike cell death caused by trauma, programmed cell death during physiological events ensures that DAMPs are contained within membranous blebs (the apoptotic bodies) and do not trigger activation of PRRs (84). The efficient coupling between apoptosis and phagocytosis avoids the development of secondary necrosis and release of DAMPs as apoptosis progresses, as well as the initiation of an inflammatory response from the immune system (84). As a result, apoptotic cell removal via phagocytosis is largely anti-inflammatory or at least immunomodulatory (67, 85), although the inflammatory responses of different types of macrophages are indeed heterogeneous (73).

In microglia, the evidences showing that phagocytosis of apoptotic cells is immunomodulatory are more tenuous than in other macrophage populations. Classic *in vitro* experiments showed that cultured microglia exposed to apoptotic cells express dampened responses to inflammatory stimuli such as bacterial lipopolysaccharides (LPS) (86, 87). *In vivo*, phagocytosis blockade induced by seizures in a mouse model of epilepsy correlated with a pro-inflammatory profile in microglia (42). Similarly, restoring phagocytosis in the aging brain using an anti-CD22 therapy reduced the microglial expression of inflammatory and disease-associated genes (74). However, the molecular mechanisms linking efferocytosis and inflammation are still unclear: is inflammation triggered by recognition of surface receptors or by downstream mechanisms related to the cargo degradation? Is it regulated at the epigenetic, transcriptional or the translational levels?

In both macrophages and microglia, these effects are at least partially mediated by apoptotic cell recognition via the complement protein C1q, whose presence turns efferocytosis anti-inflammatory (88, 89). Indeed, some of the transcriptional changes associated to efferocytosis occur while macrophages are still early in the process of phagocytosis, as shown by experiments comparing macrophages containing labeled dead cells and macrophages without apparent cargo (phagocytic and not phagocytic, respectively) (73). Similarly, posttranslational modifications related to the reduced release of the major pro-inflammatory mediator interleukin 1 beta (IL-1β) are related to the inhibition of the NLRP3 (NLR family pyrin domain containing 3) inflammasome triggered by mere contact with apoptotic cells in the absence of effective engulfment (90).

In addition to these early changes, it is also likely that the metabolic rewiring associated with apoptotic cell degradation (91) may trigger a “metabolite storm” in the phagocyte that would further contribute to regulate its function at later time points. For instance, internalization of the apoptotic cell, but not surface-to-surface interaction, triggers in macrophages another set of transcriptional anti-inflammatory changes through a chloride channel, Slc12a2, and chloride-sensing kinases (92). Time-dependent transcriptional changes have in fact been observed in cultured microglia upon phagocytosis of apoptotic cells (93). While some genes are transiently regulated at 3 h after engulfment, most are regulated in the post-degradation phase at 24 h after engulfment. This second wave of transcriptional changes is likely related to epigenetic mechanisms, as in fact chromatin remodeling related genes are upregulated in the late phagocytic microglia (93). In agreement, microglial phagocytosis is related to altered epigenetic and transcriptional profiles *in vivo*, as shown by comparing cerebellar and striatal microglia (i.e., phagocytic and non-phagocytic, respectively) (71). While this study analyzed epigenetic changes at the whole population level, it is likely that in brain areas with high basal apoptotic cell clearance, such as the cerebellum (71), the amygdala (64) or the hippocampus (37) microglia coexist in different stages related to phagocytosis. Even in these areas, it would be expected that at any given time point there would be non-phagocytic cells as well as cells engaged in different stages of engulfment, early degradation and late post-phagocytic events. This continuum of phagocytosis states is likely reflected on the epigenomic and transcriptional profiles of microglia.

### Lesson 9. Phagocytosis Alters the Phagocyte Metabolism and Function

Along with its epigenetic, transcriptional and immunomodulatory effects, phagocytosis also promotes metabolic adaptations that influence the phagocyte cellular function. In immune cells, the main metabolic pathways are catabolic (related to degradation and energy production) and anabolic (related to synthesis). Catabolic degradation of glucose starts with cytoplasmic glycolisis, which is responsible for glucose oxidation and produces pyruvate and lactate; and continues with the tricarboxylic acid cycle (TCA or Krebs cycle), which oxidizes pyruvate to produce reduced molecules (NADH, reduced nicotinamide adenine dinucleotide). NADH is also produced from the catabolism of fatty acids in the mitochondria through the beta-oxidation pathway. Next, NADH is completely degraded through the mitochondrial electron transport chain (ECT) during mitochondrial oxidative phosphorylation, to finally produce energy as ATP. Major anabolic pathways include the pentose phosphate pathway (PPP), which generates precursors of nucleotides; and the fatty acid and cholesterol synthesis pathway. The connection between metabolism and immune responses in the field of immunometabolism is very complex (94) and here we will focus on phagocyte's metabolic changes after inflammatory and phagocytic challenges.

Inflammatory stimuli trigger different types of metabolic adaptations. In pro-inflammatory conditions, macrophages need to act against infection and microorganisms and they undergo metabolic adaptations that meet the increased energetic demands while assuring cell survival. The most important change is a metabolic shift that potentiates glycolisis and downregulates oxidative phosphorylation, allowing faster, albeit less efficient, ATP production (95). In addition, several metabolic changes lead to the production of antibactericidal agents (96), such as reactive oxygen species (ROS) through increased PPP (97) and fatty acid synthesis (98); and mitochondrial ROS (mROS), through a disrupted ETC (99). Moreover, several metabolic pathways, as TCA cycle and fatty-acid synthesis are necessary for pro-inflammatory cytokine production, via HIF1α stabilization and through the fatty-acid synthesis regulator Laccase Domain-Containing Protein 1 (FAMIN), respectively (98, 100). In addition, fatty acid and cholesterol synthesis pathways also contribute to producing inflammatory mediators such as leukotriene B4 (LTB4) and isopropenoids, which bind the nuclear receptors peroxisome proliferator-activated receptors (PPARs) and the liver X receptor LXR (PLXR), respectively (101, 102). On the other hand, metabolic changes after anti-inflammatory stimuli help macrophages to resolve inflammation. In this case, upregulation of glycolisis and a proper TCA cycle contribute to the expression of an anti-inflammatory phenotype (103, 104). In addition, increased oxidative phosphorylation, together with the promotion of fatty-acid oxidation reduces the expression of pro-inflammatory cytokines (105). Thus, pro- and anti-inflammatory stimulation triggers different metabolic adaptations that contribute to modulate macrophage function.

**Figure 3:**
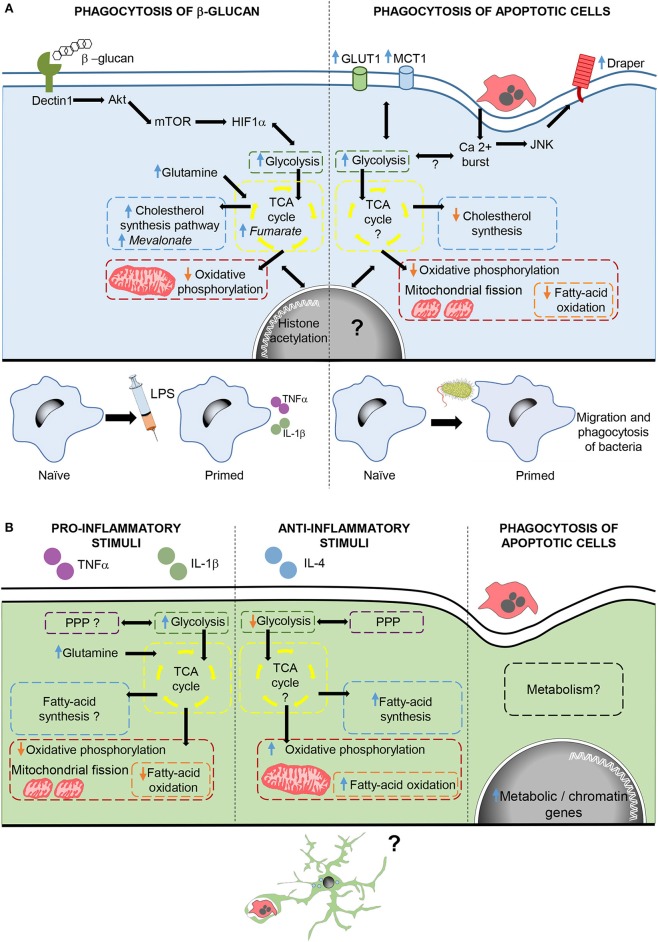
Metabolic adaptations after phagocytosis and inflammatory stimulation in macrophages and microglia. **(A)** In macrophages, phagocytosis of β-glucan activates the dectin1-Akt-mTOR-HIF1α pathway triggering metabolic adaptations required for changes in histone acetylation, which contribute to trained immunity. Phagocytosis of apoptotic cells, also triggers metabolic adaptations and increases calcium levels, which through JNK enhance the expression of the damage receptor Draper. Draper overexpression contributes to the migration and phagocytosis of bacteria by macrophages. **(B)** Microglial cells stimulated with pro- and anti, inflammatory stimuli modulate metabolism in different ways. Moreover, microglial phagocytosis increases the transcription of genes related to metabolism and chromatin remodeling, although the effect of phagocytosis in microglial metabolism and function are still unknown. In *italics*, key metabolites in trained immunity after β-glucan phagocytosis.

Phagocytosis-induced metabolic adaptations are less known. In macrophages, phagocytosis of apoptotic cells drives a downregulation of fatty acid oxidation and *de novo* cholesterol, while promoting a metabolic shift that upregulates glycolisis and reduces oxidative phosphorylation (91, 106). In fact, phagocytosis triggers mitochondrial adaptations, such as mitochondrial fission (106) and decreased mitochondrial membrane potential via mitochondrial uncoupling protein 2 (UCP2) (107), which together with increased glycolisis (91) ensure the continued uptake of corpses. In addition, increased lactate release promotes the establishment of an anti-inflammatory environment (91). However, phagocytosis-induced metabolic adaptations not only have an effect during phagocytosis, but also trigger long-lasting effects. There are several examples of how phagocytosis reprograms the function of macrophages. For instance, during Drosophila early development, naïve macrophages are insensitive to tissue damage or infection. However, upon corpse uptake macrophages become capable of migrating into damaged regions, and phagocytose bacterial pathogens, through the activation of the Jun kinase-signaling pathway and increased the expression of the damage receptor Draper (108). Similarly, phagocytosis of fungal β-glucan in mammalian macrophages drives a metabolic shift that contributes to an enhanced and nonspecific protection against infections known as trained immunity (109). In this response, macrophages upregulate glycolisis, glutaminolysis, PPP, and cholesterol synthesis, and decrease oxidative phosphorylation through the activation of the dectin-1–Akt–mTOR–HIF-1α signaling pathway. Metabolic adaptations lead to the increased production of metabolites such as fumarate and mevalonate, key for driving changes in histone acetylation and long-term epigenetic changes, which lead to increased levels of pro-inflammatory cytokines after subsequent exposure to inflammatory challenges (110–112). Thus, phagocytosis of apoptotic corpses in macrophages triggers metabolic adaptations that immediately modulate cell function and also drives a long-term reprogramming of the phagocyte (Figure 3).

In microglia, the metabolic adaptations to inflammatory stimuli are well known and similar those of macrophages, although their functional impact is less explored. Pro-inflammatory stimuli cause upregulation of glycolisis, leading to a speedy ATP generation that is crucial for the expression of pro-inflammatory cytokines (113). Other adaptations include impaired oxidative phosphorylation accompanied by mitochondrial fission (114, 115), increased glutamine entrance to the TCA cycle, suppressed fatty-acid oxidation and synthesis, and contradictory effects on the PPP (116). On the other hand, metabolic changes induced by anti-inflammatory factors in microglia are less known and show some differences compared to macrophages. Microglia exposed to anti-inflammatory stimuli maintain active both oxidative phosphorylation and PPP, and increase fatty-acid oxidation and synthesis while reducing glycolisis (116, 117). In contrast, little is known about the microglial metabolic adaptations to phagocytosis. Cultured phagocytic microglia upregulate genes related to metabolism and chromatin remodeling (93), suggesting long-term metabolic and phenotypic changes in microglia upon phagocytosis. Therefore, phagocytosis triggers a complex remodeling of the cell's metabolism and a “metabolite storm” that is likely to affect the function of microglia.

### Lesson 10. Does Phagocytosis Execute Cell Death?

**Figure 4:**
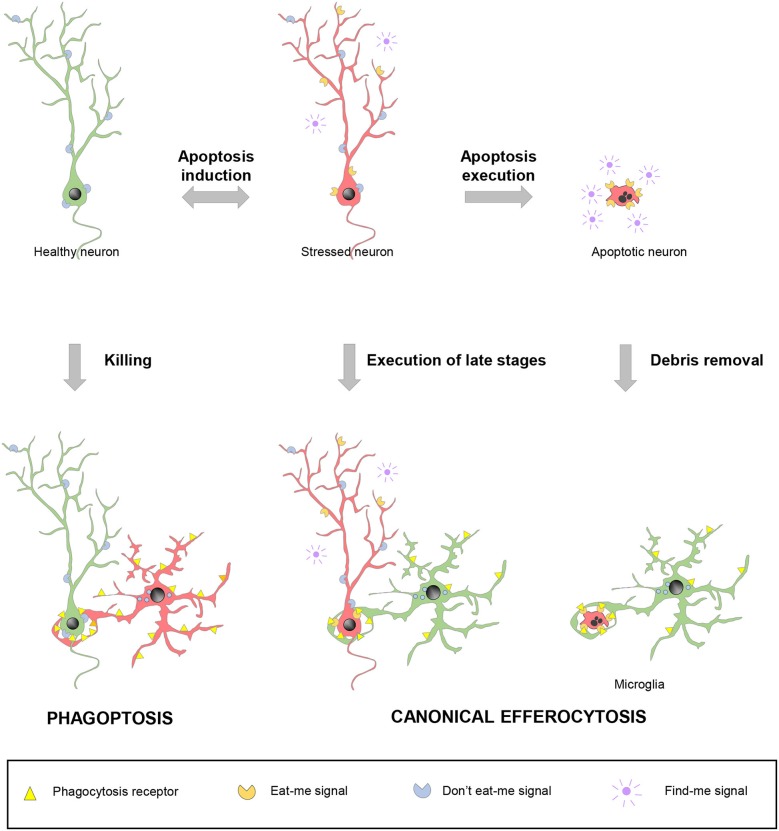
Spectrum of possibilities between physiological phagocytosis and pathological phagoptosis. Healthy neurons (green) increase their expression of released “find-me” and surface “eat-me” molecules and decrease “don't eat-me” signals under stressful situations. Stressed neurons (red) may revert to the healthy situation or follow up with the completion of the apoptotic program. In non-pathological conditions, apoptotic debris removal rapidly occurs after phagocytosis is executed by professional phagocytes, such as microglia. Under certain circumstances, stressed neurons (red) may be recognized and executed by nearby phagocytes, including microglia, before the apoptotic program has been fully engaged. In contrast to these two cases of canonical efferocytosis, in a third scenario, dysregulation of the “eat-me” and “don't-eat-me” signalization may lead microglia or other phagocytes to directly target for execution healthy, viable neurons in a process termed phagoptosis.

Since apoptosis and phagocytosis are so closely related, the last question that arises is: when is cell death precisely executed? *in vitro* experiments with apoptosis inducers clearly show that the apoptotic pathways effectively progresses to kill the target cell via proteolytic degradation of intracellular contents by caspases (118). In this conventional scenario, called-upon macrophages would simply serve to dispose of the garbage. However, the tight coupling between apoptosis and phagocytosis also presumes a second scenario, in which expeditious nearby macrophages detect the first signs of cell distress and execute the latest stages of cell death. In these two cases, canonical efferocytosis/phagocytosis is used as a homeostatic mechanism and blocking engulfment would be expected to have detrimental consequences. In a third scenario, macrophages actually select which cells die and phagocytosis causes neuronal demise, through a process named phagoptosis (119) (Figure 4).

Discerning between the three scenarios is complicated by the phagocytosis-related downstream transcriptional and metabolic changes, which in turn feedback into the surrounding cells, affecting their survival and proliferation. For instance, liver phagocytic macrophages release VEGF (vascular endothelial growth factor) to support the proliferation of neighboring cells (120). Similarly, the secretome of phagocytic microglia acutely inhibits proliferation of neural progenitors, allowing their long-term maintenance and the preservation of neurogenesis. These feedback mechanisms are likely related to the compensatory proliferation induced by killer caspases during apoptosis (“apoptosis-induced proliferation,” AiP), observed in some cell types and organisms (121). Because of these loops between death and life processes, the identification of phagoptosis must not be procedural, i.e., phagocytosis blockade increases survival, ergo, phagocytosis must kill cells. Instead, the discrimination between canonical phagocytosis and phagoptosis should be mechanistic and based on direct observation.

In macrophage biology, evidences of phagoptosis are in fact scarce and controversial. For instance, most literature claims that neutrophils die spontaneously by apoptosis after 24 h in circulation and are subsequently phagocytosed by bone marrow macrophages (67, 122). Others in contrast claim that neutrophils die by phagoptosis because phagocytosis blockade leads to more “alive” neutrophils (119), although in fact they are senescent and have impaired capabilities/migration (123). In spite of this controversy, macrophages are doubtless capable of activating the apoptosis program by direct contact through the so-called “death receptors,” such as Fas and TNFR (tumor necrosis factor alpha receptors) (124). Similarly, deletion of engulfment genes in C.elegans increases the survival of cells treated with weak apoptotic stimuli, supporting that phagocytes execute death of stressed cells (125). Microglia execute bona fide canonical phagocytosis of apoptotic newborn cells in the adult hippocampus (37). They also engage in phagoptosis during inflammatory conditions that lead to dysregulation of the “eat-me” signalization. Dysfunctional and transient expression of PS by stressed neurons leads to their recognition and execution by microglia, and their survival when microglia is not present (126). Microglia have also been reported to kill neuroprogenitor cells during cortical development, an effect that was exacerbated in mice treated with LPS (62); and Purkinje cells in the developing cerebellum, through the production of radical oxygen species (127). However, it is not clear whether in these cases cell death was executed by phagocytosis. In the end, it is likely that canonical phagocytosis and phagoptosis are two sides of a spectrum of ways to die that depends on the fine balance between the different “find-me” and “eat-me” signals in each pair of apoptotic cell/phagocyte.

In summary, phagocytosis is a powerful double-edged sword that must be kept under a tight rein, as is exemplified by two recent papers in zebrafish and Drosophila. Phagocytosis of apoptotic cells is beneficial during brain trauma, as it prevents secondary damage spread in zebrafish larvae (128). In control larvae, the initial necrotic and apoptotic cells resulting from traumatic brain injury in the optic tectum are cleared by microglia within the first 24 h. When phagocytosis is pharmacologically and genetically disturbed by targeting PS and the zebrafish ortholog of PS receptor BAI1, a larger wave of secondary cell death spread over the brain (128). In contrast, overexpression of phagocytosis receptors Six-Microns-Under (SIMU) and Draper (Drpr), homologs of Stabilin2 and MEGF10 (Multiple EGF Like Domains 10, a complement receptor), respectively, in adult *Drosophila* leads to phagocytosis of live neurons, motor dysfunction and a shortened lifespan (129). These recent papers highlight the profound physiological impact of microglial phagocytosis on the survival of their surrounding neurons both in health and in disease.

Given the powerful influence of phagocytosis on tissue homeostasis, it may seem striking that few pathologies have been related to its dysfunction. One may in fact speculate that the redundant apoptotic cell recognition mechanisms are in place to ensure that efferocytosis is effectively executed. Macrophage phagocytosis impairment has been reported mostly in the context of inflammatory and autoimmune diseases (72). For instance, macrophage efferocytosis is defective in atherosclerotic plaques, in chronic inflammatory lung diseases, and in lymph nodes of systemic lupus erythematosus patients. The involvement of phagocytosis in central nervous system diseases is, however, less explored. Mutations in MERTK lead to retinal diseases possibly linked to deficient phagocytosis of photoreceptors (130). Similarly, mutations in TREM2 or its bridge protein DAP12 are well known to cause defects in phagocytosis by osteoclasts, causing bone cysts and early dementia (Nasu-Hakola disease) (131). These mutations have been more recently associated to increased risk of AD (132) and result in deficient beta amyloid clearance in mouse models of AD (76) and reduced efferocytosis in cultured human microglia (133). In addition, mouse models and human biopsy samples have also shown impaired microglial efferocytosis during epilepsy caused by hyperactivity of the neuronal network (42). Phagocytosis therefore has a strong potential for impinging on the course of neurodegenerative diseases, as has been evidenced by blocking of the phagocytosis inhibitor CD22 in aging mouse brains to restore a microglial homeostatic profile and improve cognitive function (74).

**Figure 5:**
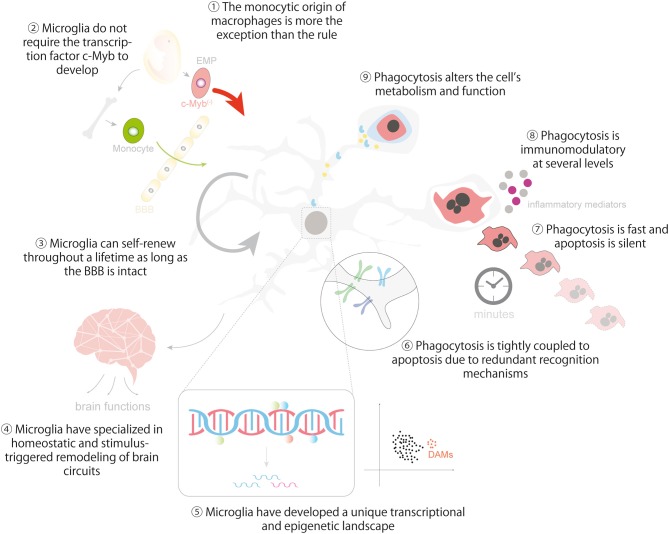
Lessons from macrophages in microglial ontogeny, identity and phagocytosis. The cartoon summarizes the main lessons that can be learnt from macrophages regarding the ontogeny and identity of microglia (1-5) and the regulation and impact of microglial phagocytosis in brain homeostasis (6-9). Lesson 10 is depicted in Figure 4. Some vector graphics were obtained from Vecteezy.com with permission.

In closing, we hope to provided enough evidence to support the idea that microglia are to some extent similar to other tissue macrophages and that important lessons can be learnt from them (Figure 5). There are remarkable similarities between microglia and macrophages in many aspects related to their origin, the establishment and maintenance of their identity, and in their dynamic epigenetic and transcriptional landscapes. They are also comparable and at the same time unique in the regulation of their phagocytosis efficiency, the cargos they engulf, and the functional consequences of phagocytosis, including metabolic adaptations, immunomodulation and its impact on the surrounding tissue. Our aspiration is that pointing out the (dys)similarities between microglia and macrophages will help to develop novel tools to harness microglial phagocytosis in the healthy and diseased brain.

## Author Contributions

All authors listed have made a substantial, direct and intellectual contribution to the work, and approved it for publication.

### Conflict of Interest

The authors declare that the research was conducted in the absence of any commercial or financial relationships that could be construed as a potential conflict of interest.
